# Factors associated with bone thickness: Comparison of the cranium and humerus

**DOI:** 10.1371/journal.pone.0283636

**Published:** 2023-03-29

**Authors:** Shimpei Goto, Keiichi Kataoka, Mutsumi Isa, Kenji Nakamori, Makoto Yoshida, Sadayuki Murayama, Akira Arasaki, Hajime Ishida, Ryosuke Kimura

**Affiliations:** 1 Department of Human Biology and Anatomy, Graduate School of Medicine, University of the Ryukyus, Nishihara, Nakagami, Okinawa, Japan; 2 Department of Oral and Maxillofacial Surgery, University of the Ryukyus Hospital, Nishihara, Nakagami, Okinawa, Japan; 3 Department of Oral and Maxillofacial Surgery, Regional Independent Administrative Corporation Naha City Hospital, Naha, Okinawa, Japan; 4 Department of Dentistry and Oral Surgery, Doujin Hospital, Urasoe, Okinawa, Japan; 5 Department of Radiology, Graduate School of Medicine, University of the Ryukyus, Nishihara, Nakagami, Okinawa, Japan; 6 Department of Oral and Maxillofacial Functional Rehabilitation, Graduate School of Medicine, University of the Ryukyus, Nishihara, Nakagami, Okinawa, Japan; The Cyprus Institute, CYPRUS

## Abstract

Cortical bone thickness is important for the mechanical function of bone. Ontogeny, aging, sex, body size, hormone levels, diet, behavior, and genetics potentially cause variations in postcranial cortical robusticity. However, the factors associated with cranial cortical robusticity remain poorly understood. Few studies have examined cortical robusticity in both cranial and postcranial bones jointly. In the present study, we used computed tomography (CT) images to measure cortical bone thicknesses in the cranial vault and humeral diaphysis. This study clearly showed that females have a greater cranial vault thickness and greater age-related increase in cranial vault thickness than males. We found an age-related increase in the full thickness of the temporal cranial vault and the width of the humeral diaphysis, as well as an age-related decrease in the cortical thickness of the frontal cranial vault and the cortical thickness of the humeral diaphysis, suggesting that the mechanisms of bone modeling in cranial and long bones are similar. A positive correlation between cortical indices in the cranial vault and humeral diaphysis also suggested that common factors affect cortical robusticity. We also examined the association of polymorphisms in the *WNT16* and *TNFSF11* genes with bone thickness. However, no significant associations were observed. The present study provides fundamental knowledge about similarities and differences in the mechanisms of bone modeling between cranial and postcranial bones.

## Introduction

Bones provide structural support for the body and serve other biological functions with regard to blood cell production and metabolism. At the macrostructural level, bone is classified into two different types: cortical (or compact) and cancellous (or trabecular) [1]. Cortical bone thickness is important for the mechanical function of bone. Cortical robusticity in cranial and postcranial bones reportedly varies among primate species, including extinct hominins. Limb cortical robusticity in primates has been studied with particular reference to mechanical loading produced by locomotion behavior and activity [2–4]. Extinct hominins, such as *Homo erectus* and *Homo neanderthalensis*, had greater cortical thickness than modern humans [5–13]. In addition, previous studies have demonstrated that cortical thickness exhibits inter-population variations in modern humans [14–18].

Age-related changes in cortical thickness, as well as in bone mineral density, have been well studied in the context of research involving osteoporosis and age-related fractures [19–26]. These studies suggested that variations in postcranial cortical robusticity can be caused by ontogeny, aging, sex, body size, hormone levels, diet, behavior, and genetics [27–30]. Genome-wide association studies conducted over the past two decades have identified genetic factors involved in osteoporosis and related traits such as bone mineral density [31]. One of these studies demonstrated that genetic polymorphisms in the *WNT16* and *TNFSF11* genes are associated with cortical thickness of the tibial diaphysis [32]. However, it is not clear whether these polymorphisms are generally associated with cortical thickness of the other long bones.

In contrast, factors associated with cranial cortical robusticity remain poorly understood. Although cranial cortical robusticity is essentially unaffected by osteoporosis, it varies according to age, sex, body size, and ancestry [33–35]. It was also hypothesized that cortical robusticity is affected in part by a systemic response to circulating hormones [10]. In addition, common genetic factors are likely to determine cortical thickness in cranial and postcranial bones. So far, however, cranial cortical robusticity has typically been studied independently of postcranial cortical robusticity. A previous study examined the correlation of cortical thickness in the cranial vault with that in the limb bones using bone specimens from multiple populations [36]. Nonetheless, the population stratification was not controlled in that study. Therefore, to what extent they are correlated remains to be determined.

Cranial and limb bones are known to differ during development [29]. Most of the cranial bones, as well as the flat bones of the face and the clavicles, are formed via intramembranous ossification, in which both cortical and cancellous bone develop from sheets of mesenchymal connective tissue. In contrast, limb bones are formed via endochondral ossification, in which bone replaces existing cartilage. Increases in limb bone length occur via interstitial growth, whereas increases in limb bone width occur through appositional growth. Bone formation by osteoblasts on the periosteal surface and bone resorption by osteoclasts on the endosteal surface determine the diameter and cortical thickness of the limb diaphysis [19, 20, 22–24]. Moreover, cranial and limb bones differ also in the responses to physiological strains [37–39]. It has been suggested that cranial bone cells do not respond to mechanical loads in the same manner as limb bone cells. However, similarities and differences in the mechanisms of bone modeling between cranial and limb bones have not sufficiently been understood.

Using computed tomography (CT) images, the present study measured cortical bone thicknesses in the cranial vault and humeral diaphysis to elucidate the factors associated with these traits. We then evaluated the correlations between cranial and humeral measurements controlling for sex, age, body size, and ancestry to identify systemic factors associated with cortical robusticity. In addition, we examined whether genetic polymorphisms in the *WNT16* and *TNFSF11* genes are associated generally with cortical thickness in limb bones and the cranial vault.

## Materials and methods

### Study subjects

CT images (slice resolution 0.98 mm for x- and y-axes; slice thickness 2.0 mm for z-axis) that included the head, trunk, and upper limbs were obtained from 504 Japanese adults (317 males and 187 females; 20–76 years of age, 57.7 years of age on average) using a positron emission tomography (PET)/CT scanner (Biograth mCT 64Slice, Siemens Healthcare, Tokyo, Japan) at the Department of Radiology, University of the Ryukyus Hospital. To confirm the effect of the resolution of CT images on the measurements, high-resolution CT images for the head (slice resolution 0.5 mm for x- and y-axes; slice thickness 0.5 mm for z-axis) were also obtained from another 25 adults (7 males and 18 females; 23–59 years of age; 36.2 years of age on average) at Naha City Hospital (Aquilion TSX-101A, Toshiba Corp., Tochigi, Japan) or Doujin Hospital (Activion16 TSX-031A/1B, Toshiba Corp., Tochigi, Japan). These images were acquired for clinical purposes. All subjects were free of congenital and systemic diseases such as cleft lip or palate and jaw deformities. From the PET/CT subjects, we also obtained saliva specimens for DNA preparation and information regarding sex, age, height, weight, and the birthplaces of their four grandparents. All subjects provided written informed consent to participate in this study. The study was approved by the Ethics Committee of the University of the Ryukyus.

### Measurement of bone thickness

**Fig 1:**
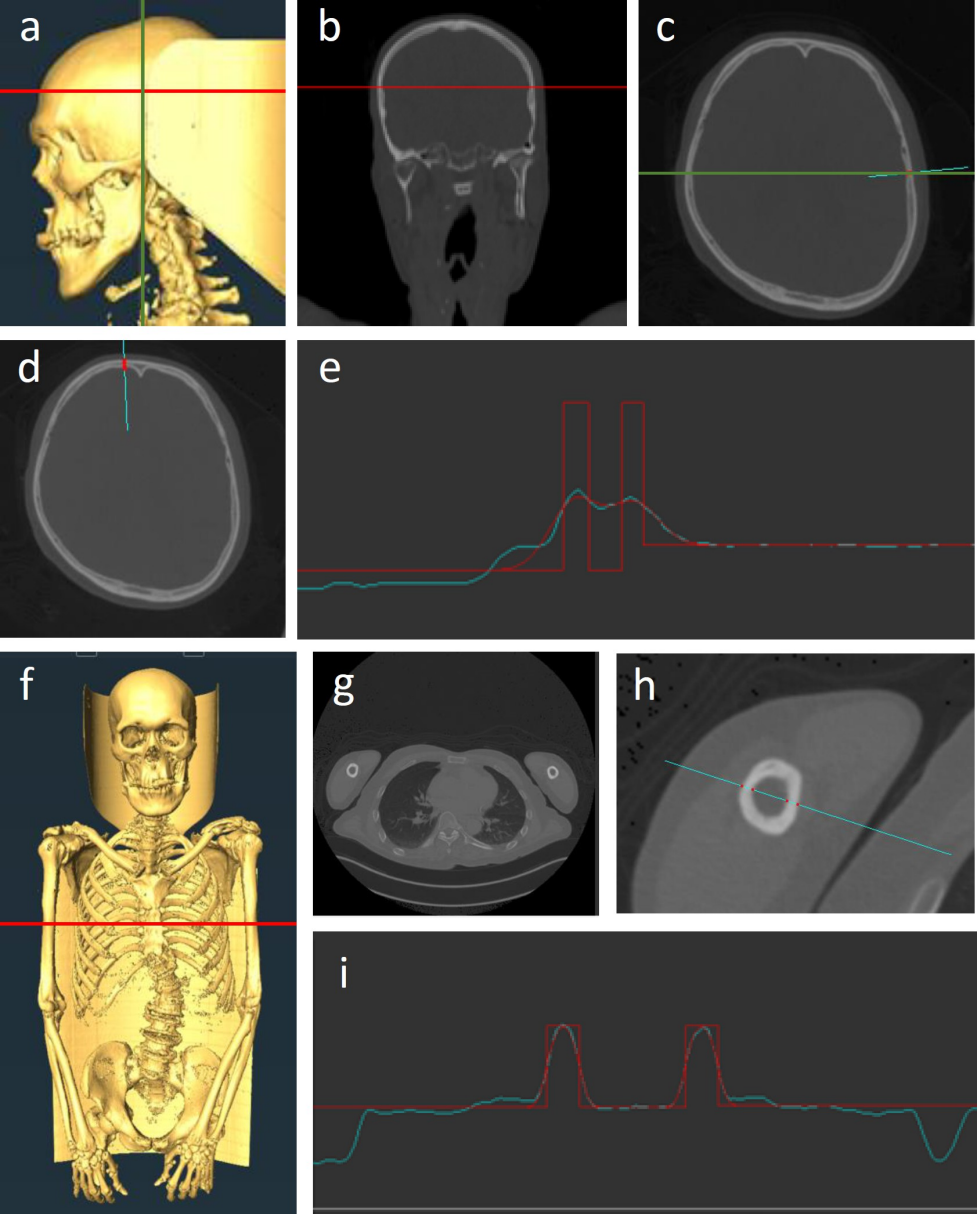
Measurement of bone thickness based on CT images. Measurement of TCVT (a-c), FCVT_full_, FCVT_cortical_ (d, e), HCT, and HBW (f-i) using Stradwin 5.4. (a-c) The green line denotes the coronal plane passing through the mandibular fossa. The red line denotes the transverse plane passing through the thinnest point of the temporal bone. (c, d, h) Measurement of cortical bone thickness along a line running through the cortex (the blue line). (e, i) Measurement is based on the intensity.

Bone thickness was measured from the CT images using Stradwin 5.4 [40]. Using both low- and high-resolution CT images, we measured the cranial vault thickness (CVT) at temporal and frontal cranial regions (TCVT and FCVT). Since the inner surface of temporal region is uneven, the thinnest points of the left and right temporal regions were identified using the coronal section passing the mandibular fossa (Fig 1A and 1B), and then TCVTs were measured at those points using the transverse section (Fig 1C). At these points, only cortical bone was contained in all the subjects. For FCVT, we measured cortical and full thicknesses (FCVT_cortical_ and FCVT_full_) at the lateral ends of slope of the frontal crest using the transverse section immediately above the frontal sinuses (Fig 1D and 1E). FCVT_cortical_ denotes the total thickness of internal and external tables. Using low-resolution CT images, humeral cortical thickness (HCT) and humeral bone width (HBW) at the shaft immediately under the deltoid tuberosity were measured on the left and right bones (Fig 1F). Using a transverse section slice, measurements were performed along the short axis of the bone section, which roughly corresponds to the anatomically mediolateral aspect of the bone. It should be noted that due to the variation in the shape of the bone section, the anatomical direction of measurement can vary. HCT denotes the total thickness of two cortical walls of the section. Although the transverse section slice was not exactly perpendicular to the humerus, the errors were negligible: even *Θ* = 5 degrees of tilt theoretically yield only a 0.38% error (1/cos*Θ*). Values for the left and right sides were averaged before the analysis. All the measurements were conducted by the first author (S. G.). Intraobserver errors were evaluated using 20 subjects of low-resolution image: intraclass correlation coefficients were 0.965 for TCVT, 0.905 for FCVT_cortical_, 0.991 for FCVT_full_, 0.981 for HCT, and 0.957 for HBW.

### Single nucleotide polymorphism (SNP) genotyping

Saliva specimens were collected and stored using an Oragene DNA self-collection kit (DNA Genotek, Ottawa, Ontario, Canada). Genomic DNA was extracted using a Gentra Puregene DNA Purification kit (Qiagen Japan, Tokyo, Japan). DNA specimens were available for 431 subjects. KASP genotyping assays (LGC genetics TW11 OLY, UK) were used to genotype two SNPs reportedly associated with tibial bone thickness in a European genome-wide association study: rs2707466 in *WNT16* and rs9525638 near *TNFSF11* [32].

### Statistical analysis

Statistical analyses were performed using jmp 14 (SAS Institute Inc., Cary, NC, USA) and IBM SPSS (IBM Japan, Tokyo, Japan). In addition to the measurements, the cranial cortical index (CCI), defined as FCVT_cortical_/FCVT_full_, and humeral cortical index (HCI), defined as HCT/HBW, were used as variables. Averages between two groups were compared using a *t*-test. Multiple regression analysis was performed to identify factors associated with the measurements or indices, including sex, age, height, weight, ancestry, and interaction between sex and age as explanatory variables. Here, sex was represented as male = 0 and female = 1. All the subjects were Japanese, and the ancestry variable denoted the number of grandparents who originated from the Okinawa prefecture (taking values 0, 1, 2, 3, and 4), which indicates the ancestry difference between the Ryukyuans and mainland Japanese. To examine the associations involving the SNPs, genotype (AA = 0, AD = 1, and DD = 2, where A and D are the ancestral and derived alleles, respectively) was also included as an explanatory variable. Correlation coefficients and partial correlation coefficients controlled by sex, age, height, weight, and ancestry were calculated between pairs of measurements/indices.

## Results

Bone thickness varied markedly among individuals. The observed average (range) values were 2.41 (1.38–5.60) mm for TCVT, 4.09 (1.56–9.00) mm for FCVT_cortical_, 5.33 (2.14–14.26) mm for FCVT_full_, 6.90 (3.42–11.73) mm for HCT, and 17.64 (12.63–23.0) mm for HBW in the low-resolution CT images (Table 1). To confirm the differences in measured values depending on modality and resolution settings, we also measured TCVT, FCVT_cortical_, and FCVT_full_ using high-resolution CT images. TCVT values in the low-resolution images (2.0 mm slice thickness) tended to be greater than those in the high-resolution images (0.5 mm slice thickness). Because TCVT was measured in the thinnest part of the temporal bone, where the inner surface is uneven, it may be difficult to capture the thinnest part using low-resolution images. We concluded that although those TCVT values may not be accurate in terms of “the thinnest part”, they could be used as an indicator of bone thickness.

**Table 1:** Summary of measurements.

Modality, resolution	Measurement	All mean ±SD (range) mm	Male mean ±SD mm	Female mean ±SD mm	Sex difference *P*
PET/CT,					
low	TCVT	2.41 ±0.73 (1.38–5.60)	2.31 ±0.64	2.58 ±0.84	4.6×10^−5^*
(n = 504)	FCVT_cortical_	4.09 ±1.03 (1.56–9.00)	3.86 ±0.88	4.43 ±1.16	6.6×10^−11^*
	FCVT_full_	5.33 ±1.54 (2.14–14.26)	4.93 ±1.37	6.02 ±1.57	2.4×10^−15^*
	HCT	6.90 ±1.62 (3.42–11.73)	7.53 ±1.50	5.84 ±1.21	8.3×10^−34^*
	HBW	17.64 ±2.10 (12.63–23.0)	18.74 ±1.54	15.76 ±1.53	3.5×10^−71^*
CT,					
high	TCVT	1.46 ±0.43 (0.87–2.28)			
(n = 25)	FCVT_cortical_	3.89±0.99 (2.02–5.76)			
	FCVT_full_	5.29±1.13 (3.12–8.56)			

TCVT: temporal cranial vault thickness

FCVT: frontal cranial vault thickness

HCT: humeral cortical thickness

HBW: humeral bone width.

**Fig 2:**
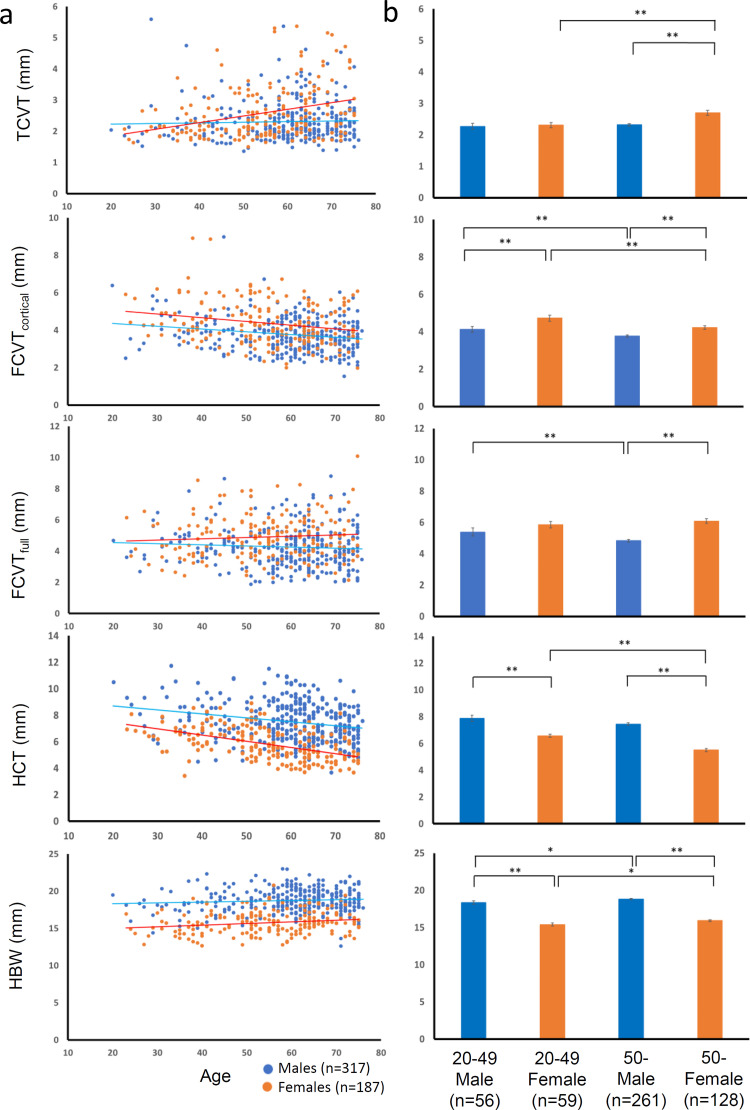
Associations of sex and age with measurements of bone thickness. (a) Plots of measurement value versus age and regression lines for males (blue) and females (orange). (b) Bar charts for sex and age groups. *P<0.05 and **P<0.01.

Males exhibited significantly higher HCT and HBW values than females. In contrast, females exhibited significantly greater CVT values than males. Fig 2A shows scatter plots for age and measurements, and Fig 2B shows graphs comparing values by sex and age. TCVT was positively correlated with age in females but not in males. However, FCVT_full_ was negatively correlated with age in males but not in females, whereas FCVT_cortical_ was negatively correlated with age in both sexes. HCT and HBW were negatively and positively correlated, respectively, with age in both sexes.

In the multiple regression analysis, the model including sex, age, ancestry, height, and weight as explanatory variables poorly explained the variation in the measurements/index for CVT (R^2^ = 0.058–0.14). CVT was significantly associated with sex, age (except for FCVT_full_), and ancestry (except for FCVT_cortical_) but not with height or weight (Table 2). In contrast, HCT and HBW were significantly associated with sex, age, height, and weight but not with ancestry (Table 3). The HCI (HCT/HBW) was associated only with sex and age. As described above, TCVT and HBW exhibited a positive association with age, whereas FCVT_cortical_, CCI, HCT, and HCI exhibited a negative association with age. The interaction term between sex and age was positively associated with TCVT and FCVT_full_ but negatively associated with CCI, HCT, and HCI (Tables 2 and 3).

**Table 2:** Multiple regression analysis of CVT.

Objective variable	Explanatory variable	*β*	SD	*t*	*P*
TCVT (R^2^= 0.072)	Intercept	2.58	0.97	2.66	**0.0081**
	Sex	0.264	0.0920	2.87	**0.0043**
	Age	0.00706	0.00278	2.54	**0.011**
	Height	-0.00559	0.00568	-0.98	0.33
	Weight	-0.000541	0.003282	-0.16	0.87
	Ancestry	0.0795	0.0253	3.14	**0.0018**
	Sex*Age	0.0188	0.0053	3.53	**4.6×10** ^**−4**^
FCVT_cortical_ (R^2^= 0.13)	Intercept	3.87	1.35	2.86	**0.0044**
	Sex	0.600	0.128	4.68	**3.7×10** ^**−6**^
	Age	-0.0164	0.0039	-4.24	**2.6×10** ^**−5**^
	Height	0.000528	0.007915	0.07	0.95
	Weight	0.00891	0.00458	1.95	0.052
	Ancestry	0.0590	0.0353	1.67	0.095
	Sex*Age	-0.00851	0.00742	-1.15	0.25
FCVT_full_ (R^2^= 0.14)	Intercept	3.14	1.99	1.58	0.11
	Sex	1.19	0.19	6.30	**6.0×10** ^**−10**^
	Age	-0.00857	0.00569	-1.51	0.13
	Height	0.00945	0.01163	0.81	0.42
	Weight	0.00184	0.00672	0.27	0.78
	Ancestry	0.183	0.052	3.52	**4.7×10** ^**−4**^
	Sex*Age	0.0247	0.0109	2.27	**0.024**
CCI (R^2^= 0.058)	Intercept	0.932	0.201	4.65	**4.3×10** ^**−6**^
	Sex	-0.0444	0.0190	-2.34	**0.020**
	Age	-0.00168	0.00057	-2.93	**0.0035**
	Height	-0.000284	0.001173	-0.24	0.81
	Weight	0.00100	0.00068	1.47	0.14
	Ancestry	-0.0190	0.0052	-3.63	**3.2×10** ^**−4**^
	Sex*Age	-0.00402	0.00110	-3.65	**2.9×10** ^**−4**^

Sex: male = 0 and female = 1

Ancestry: the number of grandparents originated from Okinawa

CCI: cranial cortical index

Bold: *P* < 0.05.

**Table 3:** Multiple regression analysis of humeral measurements.

Objective variable	Explanatory variable	*β*	SD	*t*	*P*
HCT (*R*^2^= 0.37)	Intercept	3.01	1.78	1.69	0.091
	Sex	-1.32	0.17	-7.83	**3.0×10** ^**−14**^
	Age	-0.0293	0.0051	-5.78	**1.3×10** ^**−8**^
	Height	0.0249	0.0104	2.40	**0.017**
	Weight	0.0295	0.0060	4.91	**1.3×10** ^**−6**^
	Ancestry	0.0633	0.0463	1.37	0.17
	Sex*Age	-0.0267	0.0097	-2.74	**0.0063**
HBW (*R*^2^= 0.60)	Intercept	5.45	1.86	2.93	**0.0035**
	Sex	-1.72	0.18	-9.74	**1.0×10** ^**−20**^
	Age	0.0296	0.0053	5.57	**4.1×10** ^**−8**^
	Height	0.0474	0.0109	4.36	**1.6×10** ^**−5**^
	Weight	0.0585	0.0063	9.30	**4.0×10** ^**−19**^
	Ancestry	-0.0297	0.0485	-0.61	0.54
	Sex*Age	-0.00625	0.01019	-0.61	0.54
HCI (*R*^2^= 0.16)	Intercept	46.7	10.3	4.51	**8.0×10** ^**−6**^
	Sex	-3.88	0.98	-3.96	**8.6×10** ^**−5**^
	Age	-0.245	0.030	-8.27	**1.0×10** ^**−15**^
	Height	0.0258	0.0605	0.43	0.67
	Weight	0.0390	0.0350	1.12	0.27
	Ancestry	0.365	0.270	1.35	0.18
	Sex*Age	-0.199	0.057	-3.50	**5.0×10** ^**−4**^

Sex: male = 0 and female = 1

Ancestry: the number of grandparents originated from Okinawa

HCI: humeral cortical index

Bold: *P* < 0.05.

**Fig 3:**
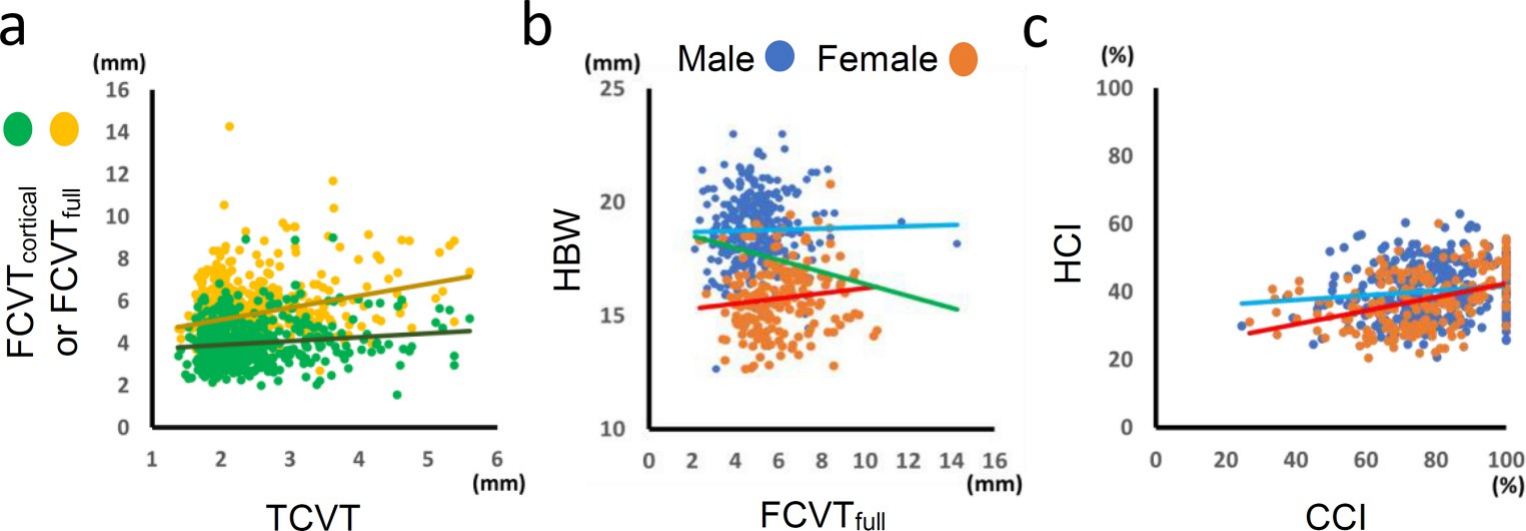
Correlations of measurements. (a) TCVT versus FCVT_cortical_ (green) or FCVT_full_ (yellow). (b) FCVT_full_ versus HBW. Plots and regression lines for males (blue) and females (orange) and regression lines for the total sample (green) are shown. (c) CCI versus HCI.

We also examined the partial correlations among measurements/indices controlling for sex, age, height, weight, and ancestry (Table 4). TCVT was correlated with both FCVT_full_ and FCVT_cortical_ (Table 4; Fig 3A). FCVT_cortical_ was correlated with FCVT_full_, and HCT did with HBW. There was no correlation between FCVT_cortical_ and HCT or between FCVT_full_ and HBW (Table 4; Fig 3B). However, a significant positive correlation was observed between CCI and HCI (Table 4; Fig 3C). When subjects were classified based on sex and age, the correlation coefficient between CCI and HCI in females was higher than that in males, and that in the younger age group (age < 50 years) was higher than that in the older age group (age ≥ 50 years) (Table 5; Fig 3C).

**Table 4:** Partial correlation coefficients between measurements.

	FCVT_cortical_	FCVT_full_	CCI	HCT	HBW	HCI
TCVT	0.11 (0.019*)	0.22 (5.3×10^-7^*)	-0.16 (3.4×10^−4^*)	-0.083 (0.064)	-0.059 (0.19)	-0.064 (0.15)
FCVT_cortical_		0.64 (7.1×10^−60^*)	0.26 (2.0×10^−9^*	0.065 (0.15)	0.036 (0.42)	0.050 (0.27)
FCVT_full_			-0.52 (6.1×10^−36^*)	-0.087 (0.051)	0.056 (0.21)	-0.11 (0.012*)
CCI				0.18 (7.7×10^−5^*)	-0.041 (0.36)	0.20 (8.1×10^−6^*)
HCT					0.11 (0.012*)	0.91 (6.0×10^−192^*)
HBW						-0.29 (3.9×10^−11^*)

Values are partial correlation coefficient (*P* value) controlled by sex, age, height, weight, and ancestry

*P < 0.05.

**Table 5:** Correlation coefficient between CCI and HCI categorized by sex and age.

	All	Age < 50	Age ≥ 50
All	0.25**	0.29*	0.21**
Males	0.12*	0.22	0.10
Females	0.39**	0.36**	0.26**

*P < 0.05

**P < 0.01.

Table 6 shows the results of genotyping for rs2707466 in *WNT16* and rs9525638 in *TNFSF11* genes that are reportedly associated with cortical thickness in the tibia [32]. Our study did not detect any significant association of these SNPs with the measurements (Table 7).

**Table 6:** Genotype and allele frequencies of the SNPs examined in this study.

SNP	Chr	Position	Gene	Location	A/D alleles†	Genotype counts (N = 431)			D allele frequency %
						AA	AD	DD	
rs2707466	7	121339035	*WNT16*	Exon 4	T/C*	3	88	340	89.1%
rs9525638	13	425554441	*TNFSF11*	8kb upstream	T*/C	226	169	36	28.0%

†Ancestral (A) / Derived (D) alleles

*The allele associated with a decreased cortical thickness in tibia (Zeng et al. 2012).

**Table 7:** Regression analysis examining the association of SNPs with bone measurements.

Objective variable	Explanatory variable	*β*	SD	*P*
TCVT	rs2707466 (*WNT16*)	0.0611	0.0765	0.43
FCVT_cortical_	rs2707466 (*WNT16*)	0.0294	0.1105	0.79
FCVT_full_	rs2707466 (*WNT16*)	0.127	0.161	0.43
CCI	rs2707466 (*WNT16*)	-0.0193	0.0160	0.23
HCT	rs2707466 (*WNT16*)	-0.163	0.145	0.26
HBW	rs2707466 (*WNT16*)	0.225	0.150	0.13
HCI	rs2707466 (*WNT16*)	-0.0161	0.0084	0.056
TCVT	rs9525638 (*TNFSF11*)	0.00149	0.05123	0.98
FCVT_cortical_	rs9525638 (*TNFSF11*)	-0.0731	0.0739	0.32
FCVT_full_	rs9525638 (*TNFSF11*)	0.0809	0.1078	0.45
CCI	rs9525638 (*TNFSF11*)	-0.0169	0.0107	0.11
HCT	rs9525638 (*TNFSF11*)	0.142	0.097	0.14
HBW	rs9525638 (*TNFSF11*)	-0.0178	0.1007	0.86
HCI	rs9525638 (*TNFSF11*)	0.00839	0.00563	0.14

SNP genotype (AA = 0, AD = 1, and DD = 2

where A and D are the ancestral and derived alleles, respectively) was examined together with other covariates (sex, age, height, weight, and ancestry).

## Discussion

### Sexual dimorphism in bone thickness

Because of sexual dimorphism in body size, males usually have a larger postcranial bone volume than females [28]. Some previous studies have shown that even after controlling for body size, males have greater bone volume and greater cross-sectional area in postcranial bones than females [41, 42]. The present study clearly demonstrated that males have greater cortical thickness and greater width in the diaphysis of the humerus than females, even after controlling for other covariates, including height and weight (Fig 2 and Tables 1 and 3). It has been suggested that skeletal sexual dimorphism is due not only to differences in sex steroid secretion between males and females but also to complex interactions between many factors, such as sex hormones, the growth hormone and insulin-like growth factor–1 pathways, and mechanical loading [43–46].

Sexual dimorphism in CVT is a controversial subject. Some studies have observed no sexual difference in CVT [16, 33, 47–49]. Other studies, however, have reported sex-related differences in CVT in particular cranial regions [14, 15, 50–60]. Generally, it seems that males have greater CVT in the posterior region, whereas females have greater CVT in the anterior region [34]. The present study, which found that females have greater CVT in the frontal and temporal bones (Fig 2 and Table 1), supports the previous general findings. In addition, our regression analysis (Table 2) showed that CVT values were not associated with body size (height and weight), in contrast to the humeral measurements. These data thus suggest that sex itself is a factor determining CVT and that it oppositely affects cranial and postcranial bones. It can be hypothesized that estrogen signaling plays an important role in the increased CVT of females. To elucidate the mechanism underlying sexual dimorphism in CVT, however, further studies will be needed.

### Age-related changes in bone thickness

Various studies have suggested that growth in the width of long bones through periosteal apposition is retained throughout the human lifespan, with age-related loss of cortical thickness via endosteal resorption occurring to a greater extent in females than males, primarily due to estrogen deficiency after menopause [24, 28, 29, 61]. Consistent with these observations, our study showed that HBW increases with age and that there is no difference in the age-related increase in HBW between males and females (Fig 2 and Table 3). The age-related decline in HCT was greater in females than males. In addition, a negative correlation between HBW and HCI indicated that the proportion of the medullary cavity increases as bone width increases (Table 4).

Only a few studies have examined age-related changes in CVT. These previous studies did not detect any significant age-related change in full CVT values in adults [33, 34]. Lillie et al. [35] reported a slight, but not significant, increase in full CVT with age and a significant decrease with age in the thickness of the inner and outer cortical tables. In the present study, we found a significant positive association between TCVT and age (Fig 2 and Table 2). In addition, multiple regression analysis demonstrated that the age-related increase in TCVT was greater in females than males and that the age-related effect on FCVT_full_ also differed between males and females. We also detected an age-related decrease in FCVT_cortical_. Age-related decreases in FCVT_cortical_ and HCT and age-related increases in TCVT and HBW suggest that the cranial and long bones share common mechanisms of bone resorption along the endosteal surface and bone formation along the periosteal surface. However, it is notable that the age-related effect on FCVT_cortical_ did not differ with sex, in contrast to long bones. Therefore, post-menopausal estrogen levels may not be responsible for the age-related decline in cortical thickness in the cranial vault.

### Effects of Okinawan ancestry on CVT

In the present study, we examined only Japanese people living in Okinawa Prefecture. The participants included individuals of Okinawan (Ryukyuan) ancestry and of mainland Japanese ancestry. Previous anthropological and genetic studies have demonstrated that the Okinawan people are genetically and phenotypically differentiated from the mainland Japanese people [62–64]. Therefore, we examined the effects of ancestry on bone thickness. As results, individuals of Okinawan ancestry showed significantly higher values in TCVT and FCVT_full_ than those of mainland Japanese ancestry. It has been shown that ancient Jomon skulls have larger CVT than modern Japanese skulls [16]. Since it has been also suggested that genetic contribution of Jomon to the Okinawans is larger than those to the mainland Japanese [65], the difference in CVT depending on ancestry may be attributed to Jomon-derived genetic variations.

### Correlation between cortical thicknesses of the cranial vault and humeral diaphysis

Few studies have examined cortical thickness of both the cranial and postcranial skeleton. A previous study focusing on the link in cortical robusticity between the cranial and limb (humerus and femur) bones reported correlations in the proportional cortical thickness (*R* = ~0.4) [36]. However, as that study used samples derived from a variety of populations, the correlations might have resulted from population stratification in the samples. In the present study, we calculated partial correlation coefficients, controlling for ancestry as well as sex, age, height, and weight as covariates. As a result, we found a low positive correlation between CCI and HCI (*R* = 0.20) (Table 4), which indicates the existence of common factors that affect cortical thickness in both cranial and postcranial bones. In addition, we observed that females and the younger age group exhibited a higher correlation coefficient between CCI and HCI than males and the older age group, respectively (Table 5; Fig 3C). This suggests that systemic factors involved in the variation in cortical robusticity play a more significant role in females than males and before reaching advanced age than after reaching advanced age.

Genetic factors are most likely associated with the systemic mechanisms that affect cortical robusticity in both cranial and postcranial bones. Molecules involved in bone formation and resorption tend to be common throughout the body. In particular, intramembranous ossification in cranial bones and appositional growth in limb bones share the same bone modeling mechanism. Therefore, genetic variations that alter the functions of related molecules are expected to have systemic effects. Meanwhile, cranial and limb bones differ in the responses to mechanical loading [37–39]. It has been hypothesized that the activity of limb bone cells depends on the strength of mechanical loading, whereas the activity of cranial cells is retained despite very low levels of mechanical loading. In the present study, we observed that CCI and HCI are highly correlated in young females (Table 5). One of the reasons for this may be that, in the older age group, there is an only small correlation, if any, between the age-related effects on HCI and on CCI. Furthermore, meles may have a greater individual difference than females in the mechanical loading on limbs, depending on physical activity.

Alternatively, responses to exogenetic stimuli might also be involved in the systemic mechanisms affecting cortical robusticity. In a study using pigs and armadillos, Lieberman [10] demonstrated that regularly exercising animals exhibited significantly higher cortical robusticity in both the cranial and postcranial bones than non-exercising controls, suggesting that cortical robusticity in cranial bones is acquired via hormones such as growth hormone and insulin-like growth factors, but not directly through mechanical loading. Although Lieberman’s observation was not replicated in a study on mice [66], levels of circulating factors such as hormones, growth factors, cytokines, and metabolites can nonetheless serve as non-genetic systemic factors [43, 45, 46].

### Association of *WNT16* and *TNFSF11* polymorphisms with bone thickness

The molecular basis of bone development, remodeling, and aging has been well studied [30, 67, 68]. Genome-wide association studies have identified hundreds of genetic loci associated with osteoporosis and related traits [31]. A previous study reported that polymorphisms in the *WNT16* (rs2707466) and *TNFSF11* (*RANKL*) (rs9525638) genes were strongly associated with the cortical thickness of the tibial diaphysis [32]. WNT16 is a positive regulator of both cortical and trabecular bone mass and structure [69–72]. TNFSF11 is a key regulator of bone remodeling and essential for osteoclast differentiation, activation, survival, and enhancement of bone resorption [73–77]. In the present study, we examined polymorphisms in *WNT16* (rs2707466) and *TNFSF11* (*RANKL*) (rs9525638) as candidate genetic factors exhibiting systemic effects. We did not observe any significant association of these polymorphisms with the measurements of bone thickness, but there were a few instances where statistical tendencies were found in our analysis (Table 7). Especially in the analysis for HCI (*P* = 0.056 for rs2707466 and *P* = 0.14 for rs9525638), we confirmed that the direction of an allelic effect was the same as the previous study analyzing tibial cortical thickness. Bone thickness is a polygenic quantitative trait, and the effect size of each genetic variant on this trait is very small. Therefore, the sample size in this study may have been insufficient to detect an effect on bone thickness. Further studies with a larger sample size are thus needed to identify systemic genetic factors affecting cortical robusticity.

## Concluding remarks

This study clearly showed that females have greater bone thickness than males in the cranial vault, in contrast to the humeral diaphysis. We also identified similarities and differences in age-related effects on cortical thickness between the cranial vault and humeral diaphysis. A positive correlation between CCI and HCI (*R* = 0.20) was observed after controlling for confounding factors, suggesting the existence of systemic factors that affect cortical robusticity. Our genetic analysis examining polymorphisms in the *WNT16* and *TNFSF11* genes did not detect any significant association between these polymorphisms and bone thickness. The present study, which adds insight into the differences in cortical robusticity between cranial and postcranial bones, enhances current understanding of the mechanisms of bone modeling.

## Supporting information

S1 Data Raw data for measurements. (XLSX)
